# Orchestration of saccadic eye-movements by brain rhythms in macaque Frontal Eye Field

**DOI:** 10.1038/s41598-023-49346-0

**Published:** 2023-12-20

**Authors:** Yeganeh Shaverdi, Seyed Kamaledin Setarehdan, Stefan Treue, Moein Esghaei

**Affiliations:** 1 Control and Intelligent Processing Center of Excellence, School of Electrical and Computer Engineering, College of Engineering, University of Tehran, Tehran, Iran; 2 Cognitive Neuroscience Laboratory, German Primate Center – Leibniz Institute for Primate Research, Kellnerweg 4, 37077, Göttingen, Germany; 3 Westa Higher Education Center, Karaj, Iran

**Keywords:** Cognitive neuroscience, Attention

## Abstract

Visual perception has been suggested to operate on temporal ‘chunks’ of sensory input, rather than on a continuous stream of visual information. Saccadic eye movements impose a natural rhythm on the sensory input, as periods of steady fixation between these rapid eye movements provide distinct temporal segments of information. Ideally, the timing of saccades should be precisely locked to the brain’s rhythms of information processing. Here, we investigated such locking of saccades to rhythmic neural activity in rhesus monkeys performing a visual foraging task. We found that saccades are phase-locked to local field potential oscillations (especially, 9–22 Hz) in the Frontal Eye Field, with the phase of oscillations predictive of the saccade onset as early as 100 ms prior to these movements. Our data also indicate a functional role of this phase-locking in determining the direction of saccades. These findings show a tight—and likely important—link between oscillatory brain activity and rhythmic behavior that imposes a rhythmic temporal structure on sensory input, such as saccadic eye movements.

## Introduction

Saccadic eye movements—frequent and ballistic shifts of gaze—are a fundamental component of the primate visual system’s ability to scan the environment^1–4^. These movements ensure the foveation of relevant aspects of the visual scene and temporally segment the visual input into a rhythm of distinct episodes. The rhythmic nature of saccades (and several other active sensing modalities, such as sniffing and whisking) has been well-documented^5–10^. But it remains to be elucidated whether this behavioral rhythm is coupled to neural rhythms (oscillatory potentials observed within populations of neurons^11–14^). This is particularly important to ensure that the temporal structure of sensory information entering visual cortex aligns with the temporal structure of neural information processing. Here, we search for such a link, with a particular focus on the non-human primate’s Frontal Eye Field (FEF).

Saccades have been shown to determine the neuronal excitability in the visual cortex by resetting the phase of neural populations^15,16^. Previous studies have also documented a directional interaction between saccadic eye movements and LFP rhythms in various brain areas, including the monkey primary visual cortex (V1) and hippocampus^15,17–24^. Saccades modulate the power of LFP rhythms within alpha, beta and low-gamma bands in the monkey primary visual cortex (V1)^15,18^. Not only the power, but the phase of rhythmic LFPs have been shown to be reset by saccades, both for human and non-human primates. These studies have demonstrated that saccades reset the phase of oscillatory activity in the visual cortex and hippocampus, particularly in the delta-theta band^17,19,20,22,23^ and further electrophysiological recordings in monkeys have shown a concentration of phases following fixation onset in a wider range of frequencies, alpha (8–14 Hz), beta (14–30 Hz), and gamma (30–60 Hz) bands in the upper bank of the superior temporal sulcus^21^. While saccades are known to be critical in determining the upcoming rhythmic activity, there is limited knowledge on how rhythmic activities modulate saccades; however, see^24,25^.

To deepen our understanding of the neural mechanisms underlying the temporal pattern of saccades, here we analyze neural recordings from the FEF, a brain area that plays a key role in guiding eye movements. To examine the potential role of the FEF's rhythmic neural activity in saccade timing, here we probe the locking of saccades to the phase of LFP oscillations recorded from macaque monkeys performing a visual foraging task.

## Results

**Figure 1:**
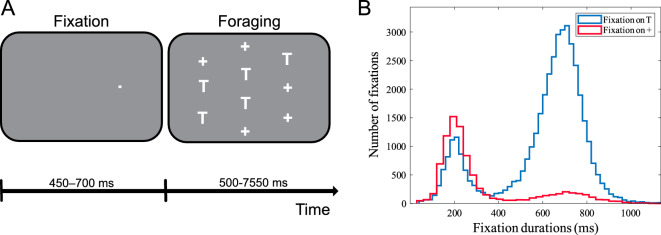
Stimulus configuration and fixation statistics. (**A**) Example stimulus arrangement in the foraging task, with five potential targets (‘T’) and five distractors (‘+’). The animal initiated the task by directing his gaze towards the fixation spot that appeared on one side of the screen. Following a delay ranging from 450 to 700 ms, an array of stimuli emerged, with five potential targets (‘T’) and five distractors (‘+’). Notably, one of these ‘T’ symbols was associated with a reward. To obtain the reward, the animal had to successfully fixate on the reward-associated ‘T’ within a 1.5° window and maintain the fixation for at least 500 ms. This action had to be completed within an 8 s timeframe from the start of the trial. (**B**) Histogram of the duration of eye fixations on ‘T’s (blue) and ‘+’s (red), excluding the rewarding fixation.

Two rhesus monkeys were trained to perform a foraging task (Fig. 1A), searching among five potential targets (‘T’ shape) and five distractors (‘+’ shape) randomly arranged on the screen for the one ‘T’ associated with a reward. When an animal foveated (‘fixated’) the reward-associated ‘T’ (independently selected in each trial) for at least 500 ms, he received a juice reward. Notably, there was no cue provided to indicate the location of the reward-associated ‘T’ and therefore the animal had to sequentially fixate on each of the ‘T’ shapes to find the one associated with the reward (see the “Methods” section). In case the animal successfully fixated on the reward-associated ‘T’ within 8 s from the trial start, he received the reward, otherwise the trial aborted without a reward.

Fixations on distractors were infrequent (17.5%, 8,933 out of 50,854) as well as substantially and significantly shorter (320 ± 221 ms (mean ± SD), *p* < 0.0001, permutation test; n = 10,000) than fixations on potential targets (602 ± 204 ms).

LFPs and spiking activity were recorded from both animals in 61 separate sessions. There were on average 373 correctly performed trials (ending with a saccade towards the reward-associated ‘T’) in each session. In the current study, we focused on saccades which were made towards ‘T’s. The duration histogram of fixations following these saccades is plotted in Fig. 1B, showing that fixations on ‘T’s follow a bi-lobed (Davies–Bouldin index^26^) distribution with peaks centered at 205 and 697 ms. This suggests that the animals’ eye-fixation after making a saccade to a target lasted either a short (~ 200 ms) or a long time (~ 700 ms). The fact that the majority of these fixations lasted more than 500 ms (33,065 of 41,921 (78.8%)), further indicates that the animals were correctly following the foraging paradigm.

**Figure 2:**
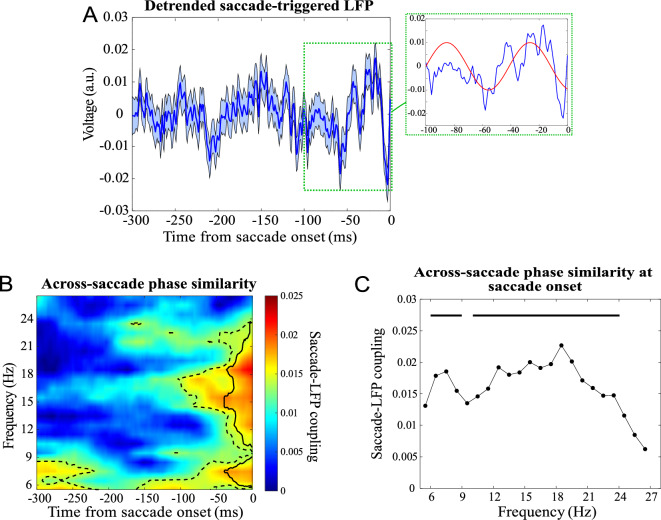
Dependence of the onset of saccades upon the phase of ongoing LFP oscillations. (**A**) Saccade-triggered LFP across all saccades (error bars show the standard error of mean), with a zoomed-in version for [-100,0] ms showing a ~ 17 Hz oscillatory component (red curve) during the interval. (**B**) Across-saccade phase similarity; dashed and solid border lines indicate significant phase clustering values (Rayleigh test, *p* < 0.05) before and after controlling for multiple comparisons (using FDR correction), respectively. (**C**) Across-saccade phase similarity computed at the saccade onset for each frequency band (solid lines show significant phase clustering with a threshold of *p* < 0.05 for each frequency band).

To examine if the timing of the eye movement initiation follows an oscillatory pattern, we asked if they were coupled to the LFP signals. To this end, the average of the LFPs preceding the onset of saccades (named saccade-triggered LFP) was calculated after removal of the transient preparatory component (see “Methods” section for details) (Fig. 2A). This saccade-triggered LFP is clearly indicative of a locking of saccades to the preceding oscillatory fluctuations of the LFP, especially within the beta band (17–21 Hz). To quantify the alignment of saccade onsets to the phase of the preceding ongoing neural oscillations, we calculated the phase-locking value (PLV) at the start of the saccades across different frequency ranges. LFPs were filtered into sweeping (step size: 1 Hz) frequency bands of 3 Hz width and a lower boundary ranging from 4 to 25 Hz. Next, we extracted the instantaneous phase of the filtered LFPs for the interval [− 300, 0] ms before the saccade onset time and calculated the similarity of phases for each time–frequency pair. Figure 2B shows the across-saccade phase similarity for all time–frequency pairs, indicating that saccades are phase-locked to the LFP oscillations for most frequencies lower than 22 Hz as early as ~ 100 ms before the saccade onset (Fig. S1 shows each animal’s data separately). Figure 2C plots the frequency-resolved PLV at the time of saccade onset (x-axis indicates the middle of each frequency band), showing that saccade onsets were maximally locked to the frequency band of 17–20 Hz (p < 0.01, Rayleigh test; corrected for multiple comparisons using FDR). We further compared the phase locking value computed at saccade onset with that computed 250 ms before saccade onset (Fig. S2), showing a significant PLV difference between the two time points along 10–20 Hz. The circular histogram of the instantaneous LFP phase at the saccade onset for the frequency band with maximum phase-locking (17–20 Hz; Fig. S3), indicates how saccades are phase-locked to the trough (~ 220°) of beta oscillations. An extension of the analysis beyond the pre-saccade period showed a decrease of beta-band phase synchrony as time progressed after saccade onset (Fig. S4). This suggests that the significant beta-band phase synchrony we have observed is indeed associated with pre-saccadic brain activity, rather than being a consequence of saccadic eye movements.

Results remain consistent across different filter settings (Fig. S5). These data show that the onset of saccades is typically aligned to the phase of the oscillatory LFP, i.e. follow a rhythmic pattern.

**Figure 3:**
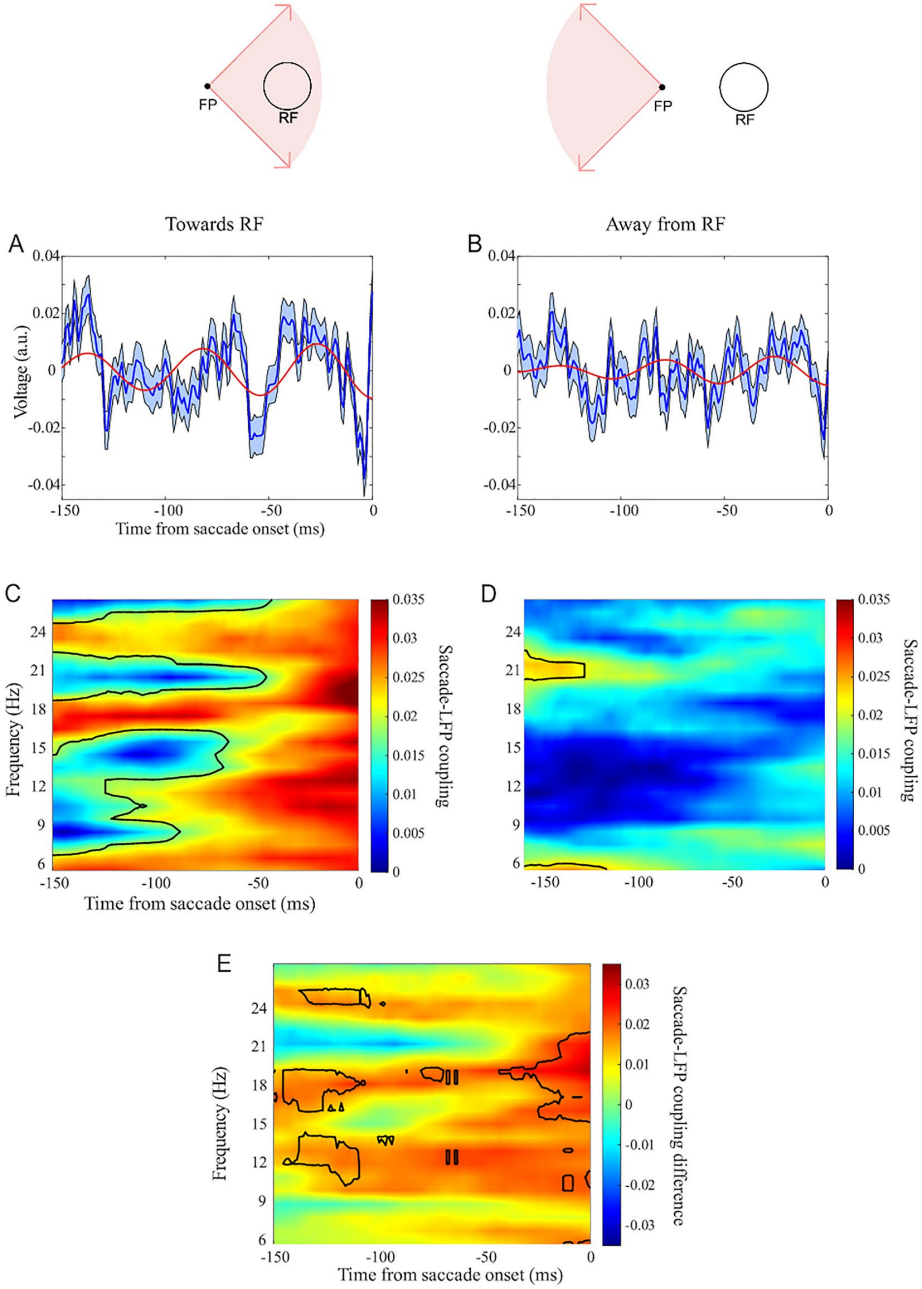
Direction dependence of across-saccade phase similarity. (**A**) Saccade-triggered LFP for saccades towards RF and (**B**) away from RF (error bars show the standard error of the mean). Red curves show the saccade-triggered filtered LFP (18–21 Hz band-pass filtering). (**C**) Across-saccade phase similarity for saccades towards RF and (**D**) away from RF (Black border lines indicate significant clusters of values (Rayleigh test, *p* < 0.05) after controlling for multiple comparisons using FDR correction.) (**E**) Direction dependence of the across-saccade phase similarity, computed by comparing saccades towards and away from the RF (subtracting values in map D from map C). Black boundaries demarcate significantly different PLVs (*p* < 0.05, permutation test, n = 1000).

To examine the functional relevance of the locking of saccade onsets to the LFP phase, we next categorized the saccades based on their direction relative to the response field (RF) of the recorded neuron/population; saccades towards the RF ([− 45° + 45°]) and saccades away from the RF ([135° 225°]) (Fig. 3). These two categories were behaviorally equivalent since the animal was not aware of the location of the recorded neuron’s RF. Figure 3A,B show the saccade-triggered LFP for these saccades (towards and away from RF, respectively), visually clarifying how the toward-RF saccades follow a saccade-locked beta component, whereas away-RF saccades lack such phase-locking (see the filtered beta components overlaying the original signals). The across-saccade similarity of instantaneous LFP phases were then calculated within each group of saccades and plotted across time ([− 150, 0] ms around the saccade onset) along different frequencies (Fig. 3C,D for saccades towards and away from RF, respectively). Saccades towards the RF exhibited a significant phase similarity starting from at least 150 ms before the saccade onset, while no such phase similarity was observed for saccades away from the RF (Fig. 3C,D). We also observed a gradual decrease in phase similarity as saccades moved further away from the RF (see Fig. S6). Also we plotted the difference map between towards and away saccades with the permutation test results (Fig. 3E). To rule out the possibility that this difference was due to variations in LFP’s signal-to-noise ratio, we analyzed the pre-saccadic spectral power in ‘towards RF’ and ‘away from RF’ saccades (see Fig. S7A). Interestingly, we found that the spectral power in the beta range was lower for saccades towards the RF, suggesting that differences in signal-to-noise ratio did not account for the observed difference in phase-locking (see^27^ for similar results). To control for the possibility that the PLV differences are driven by the power difference, we conducted a simulation to investigate the relationship between phase-locking values and signal-to-noise ratio (Fig. S7B). The dependence of saccade-LFP coupling was not attributable to differences of saccade amplitude between saccade towards RF and saccades away from RF (Fig. S8). These results suggest that not only the timing of saccades is aligned to the phase of LFPs, but also that this alignment changes as a function of the spatial relationship between the saccade target and the RF, indicating a role of oscillatory neural activities in determining the direction of saccades relative to a neuron’s RF. These findings indicate that oscillatory neural activity has a distinct role in regulating the generation of saccades and their direction relative to the RF, shedding light on the neural activity patterns underlying eye movements.

## Discussion

A fundamental aspect of the primate visual system is the existence of a retinal fovea, covering a small fraction of the visual scene at a much higher resolution than is available for the vast rest of the visual field. To ensure that complex visual scenes—with potentially multiple, spatially separated points of interest—are optimally scanned, the primate visual system combines the foveal specialization with frequent and ballistic shifts of gaze to sequentially foveate these points of interest for a detailed visual scrutiny. These saccadic eye movements temporally segment the visual input into a rhythm of distinct episodes. It is therefore of fundamental importance for an understanding of primate visual information processing to elucidate the relationship between the behavioral rhythm of eye movements and the brain’s rhythms of information processing.

Here, we show that the timing of saccades is phase-locked to the low frequency (9–22 Hz) oscillations of local field potentials in the Frontal Eye Field of macaque monkeys, documenting a precise entrainment of saccades to neural oscillations in primate cortex.

Our data demonstrate a significant phase similarity in LFPs within the 100 ms period prior to saccades. Interestingly, this time window aligns with the time it takes (~ 100 ms) to initiate and execute a saccade, even in everyday tasks like reading^25,28^. Thus, our results not only support the notion that the brain's processing of visual information and saccade planning are tightly coordinated, but also suggest that the phase-locking of saccades during this critical 100 ms period may play an important role in the initiation and execution of saccades.

FEF has been shown to play a crucial role in both motor planning (for eye movement) and visual attention, however it remains unclear whether the distinguished beta phase locking observed in our study is related to the motor or the attentional role of FEF. Our observation of saccades being locked to beta LFP oscillations in the FEF, confirms previous studies indicating the relevance of beta oscillations in motor areas. The beta frequency band is a well-known feature of LFPs recorded from the primary motor cortex and the supplementary motor area^29,30^, and has been shown to be crucial for motor planning, movement execution, and motor inhibition^31,32^. This suggests that the involvement of beta oscillations in saccadic eye movements may reflect the motor planning and execution processes involved in generating eye movements. Further studies are needed to determine the potential functional significance of the beta phase locking to the guidance of pre-saccadic visual attention.

There are indications that theta rhythmic neural activities may also have a functional role in driving saccadic eye movements^33^. We speculate that beta-driven saccades may be further cross-frequency coupled to theta rhythms. This implies that beta rhythms are stronger during certain phases of theta oscillations, and weaker during other phases, potentially driving saccades relative to the theta phases. This emphasizes the fact that coupling of saccades to a certain rhythmic neural activity does not imply a rhythmicity of saccades within that frequency and raise intriguing questions about the relationship between different frequency bands and their functional roles in saccade generation. Further investigation of such cross-frequency coupling may shed light on the neural mechanisms underlying the coordination of selective visual processing and movements of the eye towards the focus of attention.

An investigation of the interaction between the ongoing oscillatory activity and saccades is only possible with a proper filtering of the oscillatory components. This becomes a particular challenge in presence of non-stationary signal components, such as those related to transient preparatory activities, causing a potential artifact distorting the conclusions drawn. In our study, we took extra care to ensure the reliability of our observations by a polynomial estimation of the transient neural activity component preceding each saccade. We are confident, that this approach has removed not only the average transient component within a neural population, but also to have captured the saccade-by-saccade variation of this component, enabling an accurate estimation of the intrinsic rather than induced oscillatory activities relevant to saccades.

Regarding the directionality of the beta-saccade interaction, we consider the following three scenarios; (1) Beta—> saccade and saccade—> beta: while beta oscillations preceding the saccade determine the saccadic occurrence, saccades also drive the oscillatory activity during the post-saccadic interval. (2) Saccade—> beta; It is only the saccades that drive the oscillatory activities; the pre-saccadic LFP pattern (that is observed to be linked with the saccade) is only a leakage of oscillations following the saccade. (3) Beta—> saccade; It is only the beta rhythm that drives saccades and the post-saccadic LFP oscillations that are observed to be aligned to the saccade are a simple continuation of the pre-saccadic beta rhythm that has also aligned the saccade. Notably, we exclude scenario #2 after considering only the pre-saccadic LFPs and replacing the post-saccadic LFP with noise (when band-pass filtering), thereby avoiding the possibility of leakage from the post-saccadic interval. Our findings indicate that beta oscillations play a driving role in saccades. However, the question of whether saccades reciprocally influence the beta oscillations during the post-saccadic interval remains open for exploration in future studies.

In summary, our findings illuminate a precise temporal coordination between saccadic eye movements, which sample the visual environment in temporal ‘chunks’, and the brain’s rhythms of information processing. These findings underscore the functional importance and the tight coupling of rhythms in behavior and neural processing. They also open the possibility that such coupling in the visual system can serve as a model for the potential coupling of a range of other rhythmic behaviors with neural rhythms.

## Methods

We used data from the study conducted by Mirpour et al. which are accessible through https://osf.io/und8y/. All experiments were approved by the Chancellor’s Animal Research Committee at the University of California–Los Angeles to comply with the guidelines established in the Public Health Service Guide for the Care and Use of Laboratory Animals. LFPs and spikes were recorded from two behaving male rhesus macaques (8–12 kg). Surgical and implantation procedures as well as localization of the region of interest (FEF) are reported in previous papers^34,35^.

### Behavioral task and electrophysiological recording

Two monkeys were trained on a visual foraging task. They started a trial by fixating on a spot that appeared on one side of the screen (Fig. 1A). After a delay of 450–700 ms, an array of five potential targets (‘T’) and five distractors (‘+’) was presented (each one with the size of 1.2° * 0.8°), with one over the fixation spot. One of the ‘T’s was associated with a juice reward, such that if the monkey fixated on it within 1.5° and remained fixating for 500 ms within 8 s from the start of the trial, the animal would get the reward. The stimuli were arranged so that when the monkey looked at one stimulus, the RF of an FEF neuron was likely to overlap one other stimulus. The stimuli locations, including the target, were randomly assigned among the 10 spatial locations on each trial. Extracellular single-unit activity and LFP were recorded from the anterior bank of the arcuate sulcus in the FEF using tungsten microelectrodes, with guidance from MRI coordinates. The original recordings were referenced to the guide tube, and no further referencing was used for the analyses reported in the paper. Verification of the electrode placement within FEF was confirmed by evoking saccades through microstimulation, employing current intensities of up to 50 μA. Microstimulation was administered during a blink task^36^, involving a 70-ms train of biphasic pulses (negative first, 0.2 ms width/pulse phase) delivered at a frequency of 330 Hz. Recorded neurons were considered for inclusion in the study if they exhibited increased activity during any of the visual, delayed, or motor stages of the memory-guided saccade (MGS) task and if this activity significantly exceeded the response observed during fixation on the fixation point. Consequently, fixation neurons were excluded from the study. The size and location of each neuron’s RF were mapped using an automated MGS task that covered nine and subsequently 25 locations (further details can be found in^37^). Nine probe points, organized into a 3 × 3 matrix within the contralateral visual field, were employed to investigate the RFs. This initial probing aimed to approximate the general location and size of the RF. Subsequently, based on the data obtained from this preliminary probing, a more precise 25-point array was configured using a 5 × 5 matrix. Following this refined procedure, the boundaries of the RF were delineated with a precision level of ∼ 0.5°.

Neurons were excluded from the study if their RFs were so large that they would encompass two stimuli in the array. RF centers ranged from 2.8° eccentricity to 15° eccentricity, and RF sizes ranged from 1.25° to 6.5° radius in the horizontal direction and 1.25° to 4° radius in the vertical direction^35^.

LFPs and single units were recorded from 61 distinct FEF sites from 2 animals while they performed the foraging task. In each session, correctly performed trials (ending with a saccade towards the reward-associated ‘T’) were selected.

### Data analysis

Scripts were programmed in MATLAB (MathWorks, Inc.). The total of 60,857 and 10,150 fixations were made onto ‘T’s and ‘+’s, respectively. We performed a permutation test to examine whether there was a significant difference between the duration distribution of the two fixation types. To this end, we selected fixation-to-‘T’s before the rewarding saccade (41,921) and compared their duration to that of the fixation-to-‘+’s. We selected 8,933 out of 41,921 data from the fixations landing on ‘T’s to make an equal data size of both fixation categories. Then we shuffled them with the ‘+’s fixation durations data and divided them into two equal sets with the same proportion of each fixation. We performed this shuffling for 100 iterations, calculated the mean of each category in each iteration, subtracted the means, and saved those values. The random selection of data was also made 100 times. So, the distribution of 10,000 values of the mean’s difference was created. We compared this distribution with the main difference of the distributions’ mean. The main difference was greater than 0.9999 of the distribution, suggesting that the fixations on ‘+’s were significantly (*p* < 0.0001, permutation test; n = 10,000) and substantially shorter (320 ± 221 ms (mean ± SD)) than fixations on ‘T’s (602 ± 204 ms).

To test for the bipolarity of the fixation durations distribution (Fig. 1B), we used the Davies–Bouldin index. We created a clustering evaluation object containing data to evaluate the optimal number of data clusters. The clustering was performed using the Gaussian mixture distribution algorithm and evaluated with the Davies-Bouldin index (DBI) values as a criterion. By calculating DBI for 1 to 10 clusters, the optimal number of clusters was 2, suggesting that the histogram has a bi-polar distribution. To study the neural activity related to saccades, LFP signals within [− 300, 0] ms the saccade onset was analyzed. To avoid transient neural activity evoked by preceding saccades, we focused on those saccades that were at least 700 ms apart from the previous saccade. Finally, 16,279 LFP signals (within the [− 300, 0] ms window from the saccade onset) were selected for analysis. A notch filter was applied to the LFP signals to remove the 60 Hz power line interference. Saccade-triggered LFPs were then calculated based on frequencies above 5 Hz. For filtering the signals, we used the ‘bst_bandpass_fft’ function from the Brainstorm MATLAB toolbox^38^ (freely available for download under the GNU general public license (http://neuroimage.usc.edu/brainstorm)). This function employs an FIR filter (implemented using the ‘fir2’ function from MATLAB’s Signal Processing Toolbox) with its phase slope equalized to zero (making the filter zero-phase lag), filter order of 1002, using a Hamming window. For more details, we refer to the documentation of the Toolbox^39^ and a control simulation study we have performed (Fig. S9). We did not apply any temporal smoothing when calculating the time–frequency maps. When visualizing the maps, MATLAB’s built-in interpolation routine “pcolor” was applied.

We next removed a fourth-degree polynomial trend from each saccade separately. Figure S10 shows the saccade-triggered LFP before and after detrending. Normalization of the saccade-triggered LFPs was performed saccade-wise, by calculating the z-score of the signals (mean = 0 and standard deviation = 1, calculated based on the interval − 300 to 0 ms relative to saccade-onset).

### Extraction of the phase of LFP signals

### Across-saccade phase similarity

To assess the phase-locking of saccadic eye movements to LFP oscillations, we calculated the similarity of the LFP phase at each time–frequency point preceding and relative to the saccade onset. To this end, the LFPs were filtered within different frequency bands from 4 to 25 Hz, using windows of 3 Hz width in steps of 1 Hz (i.e., 4–7 Hz, 5–8 Hz, etc.). To avoid a possible edge effect in the analysis period, we performed noise padding before the filtering, by adding 500 ms long randomly generated pink noises at the end of each LFP signal, i.e., band-pass filtering was performed on 1,000 ms long signals (consisting of 500 ms original LFP + 500 ms long pink noise). Adding pink noise, rather than simple zero-padding ensured avoiding non-stationarities in the signal by maintaining the amplitude distribution of the signal in time. To ensure the similarity of the amplitude distribution between the noise and the original signal, we first scaled each signal to [− 1, 1] and then z-scored them afterwards. We generated 100 random noises for each LFP signal. For each repetition of noise-padding to each signal, the instantaneous LFP phase was extracted using the Hilbert transform at each time–frequency pair preceding the saccade onset. Then, the circular average across phases of the 100 repetitions was calculated according to the following formula:

$$phase\left( {\tau , f} \right) = angle\left( {\frac{1}{N}\mathop \sum \limits_{j = 1}^{N} e^{{i\theta_{j} \left( {\tau , f} \right)}} } \right)$$

where ($\tau ,$* f*), *N*, and $\theta$ are a given time-frequency pair, number of repetitions, and the extracted phase, respectively. This step was performed for each of the 16,279 pre-saccadic LFP signals. Finally, the similarity between the obtained phases (across-saccade phase similarity) was calculated across saccades using the following equation:

$$Across-saccade\, phase\, similarity\left( {\tau ,f} \right) = \frac{1}{N}\left| {\mathop \sum \limits_{j = 1}^{N} e^{{i(phase_{j} \left( {\tau , f} \right))}} } \right|$$

where *N* is the number of saccades. The higher the resultant value, the stronger the saccade-LFP phase coupling for a given time-frequency pair.

To check the significance of the phase similarity at each time-frequency pair (based on the value of across-saccade phase similarity), we used the Rayleigh test. The p-values were corrected for multiple comparisons using FDR correction.

### Labeling the saccades based on their direction relative to RF

We labeled the saccades based on their angle relative to the RF of single units: saccades towards the RF ([− 45° + 45°]) and saccades away from the RF ([135° 225°]) (Fig. 3). To this end, for each recorded neuron we checked the direction of each saccade generated relative to the neuron's RF. This resulted in the labeling of 11,248 and 12,086 saccades to towards and away from RF, respectively. To reach a fair comparison between the two categories’ phase similarity maps, we computed the phase similarity map for the saccade away from RF case, based on a randomly selected subset of saccades with an equal size to the number of saccades towards the RF (11,248). To check for the significance of the difference between towards and away saccades, we performed a permutation test. We shuffled the phases of saccades towards and away from RF and made two categories with 5,624 random phases from saccades towards RF and 5,624 random phases from saccades away from RF. For each repetition, we computed the PLV for both categories and saved the value of difference between them. This process was repeated 1000 times, generating a distribution of PLV differences that reflects what we expect under the null hypothesis (no significant difference). The *p* value was then calculated by comparing the original PLV difference to the distribution of PLV differences obtained through shuffling. Specifically, we assessed what proportion of values in this distribution were larger than the observed PLV difference.

### Supplementary Information

Supplementary Figures.

## Data Availability

The datasets analyzed during the current study are available in the OSF repository, https://osf.io/und8y/.
