# Publisher Correction: Orchestration of saccadic eye-movements by brain rhythms in macaque Frontal Eye Field

**DOI:** 10.1038/s41598-024-53479-1

**Published:** 2024-02-13

**Authors:** Yeganeh Shaverdi, Seyed Kamaledin Setarehdan, Stefan Treue, Moein Esghaei

**Affiliations:** 1 Control and Intelligent Processing Center of Excellence, School of Electrical and Computer Engineering, College of Engineering, University of Tehran, Tehran, Iran; 2 Cognitive Neuroscience Laboratory, German Primate Center – Leibniz Institute for Primate Research, Kellnerweg 4, 37077, Göttingen, Germany; 3 Westa Higher Education Center, Karaj, Iran

Correction to: *Scientific Reports* 10.1038/s41598-023-49346-0, published online 20 December 2023

The original version of this Article contained errors in Figure 3, where the right image in the top panel did not display correctly.

The original Figure 3 and accompanying legend appears below.

**Figure 3:**
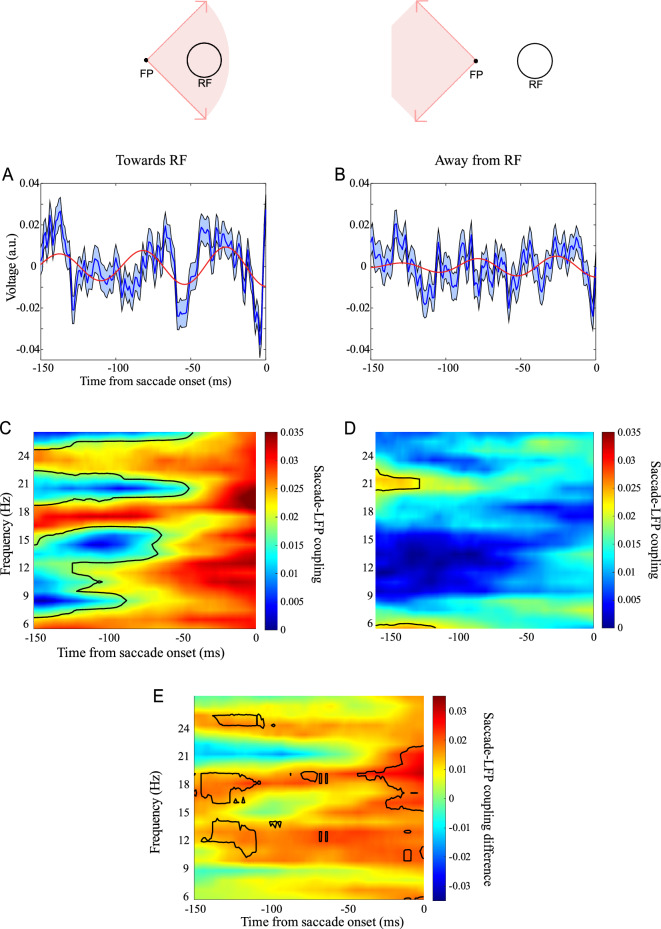
Direction dependence of across-saccade phase similarity. (**A**) Saccade-triggered LFP for saccades towards RF and (**B**) away from RF (error bars show the standard error of the mean). Red curves show the saccade-triggered filtered LFP (18–21 Hz band-pass filtering). (**C**) Across-saccade phase similarity for saccades towards RF and (**D**) away from RF (Black border lines indicate significant clusters of values (Rayleigh test, *p* < 0.05) after controlling for multiple comparisons using FDR correction.) (**E**) Direction dependence of the across-saccade phase similarity, computed by comparing saccades towards and away from the RF (subtracting values in map D from map C). Black boundaries demarcate significantly different PLVs (*p* < 0.05, permutation test, n = 1000).

The original Article has been corrected.

