# Supplementary material for: Orchestration of saccadic eye-movements by brain rhythms in macaque Frontal Eye Field
Source: Sci Rep. 2023 Dec 20;13:22725. doi: 10.1038/s41598-023-49346-0 (PMC10733338; doi:10.1038/s41598-023-49346-0)
Supplement: Supplementary file 1 — Supplementary Figures. [file 41598_2023_49346_MOESM1_ESM.pdf]

Supplementary Material for

Orchestration of saccadic eye-movements by brain rhythms in macaque Frontal Eye Field

Yeganeh Shaverdi, Seyed Kamaledin Setarehdan, Stefan Treue, Moein Esghaei

This file includes:

Figs. S1 to S10

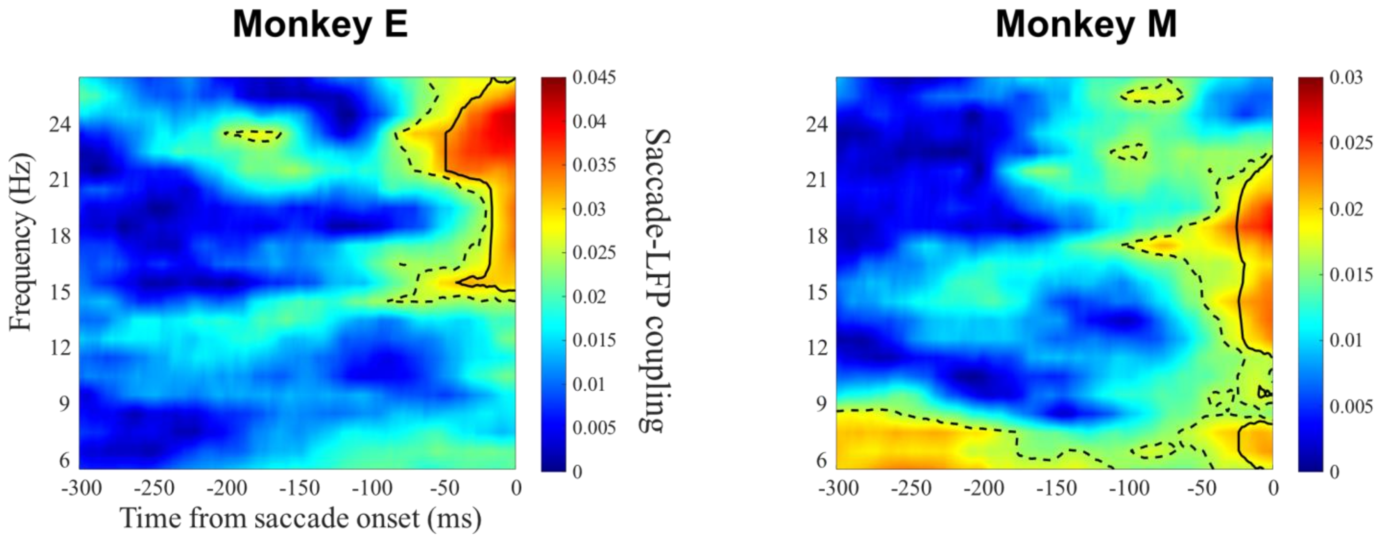

Figure S1. Across-saccade phase similarity for each of the two monkeys. Dashed and solid border lines indicate significant phase clustering values (Rayleigh test, p<0.05) before and after controlling for multiple comparisons (using FDR correction), respectively.

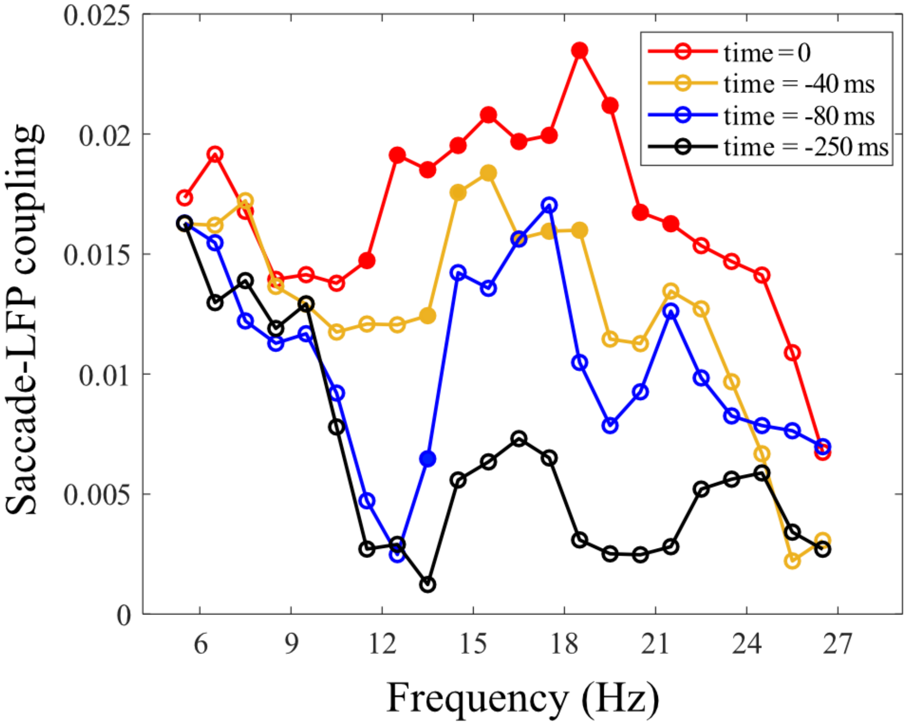

Figure S2. Permutation test between the PLV at 250 ms before saccade onset and saccade onset (red), 40 ms (orange), and 80 ms (blue) before saccade onset (n=10,000). Frequency bands denoted by filled circles indicate a significant difference of PLV with that of the -250 ms (p<0.05). To perform the permutation test, we randomly selected half of the instantaneous phases (each for an individual saccade) at 250 ms before onset and scrambled them with the corresponding saccade phases from the three time points closer to the saccade onset. We repeated this procedure for 10,000 times and each time computed the PLV for both categories and saved the difference value between them. This generated a distribution of PLV differences that reflects what we expect to observe under the null hypothesis (no significant difference). The p-value was then calculated by comparing the original PLV difference (before any scrambling) to the distribution of PLV differences obtained through permutation. Specifically, we assessed what proportion of values in this distribution were larger than the observed PLV difference.

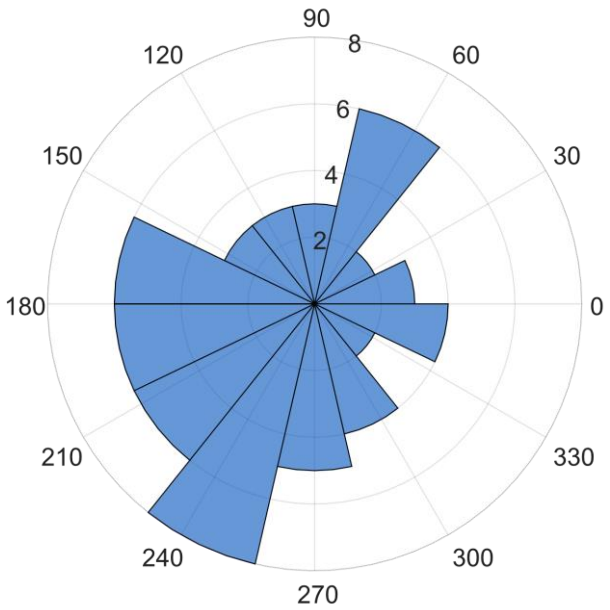

Figure S3. The circular histogram of the instantaneous LFP phase at the saccade onset within the frequency band of 17-20 Hz.

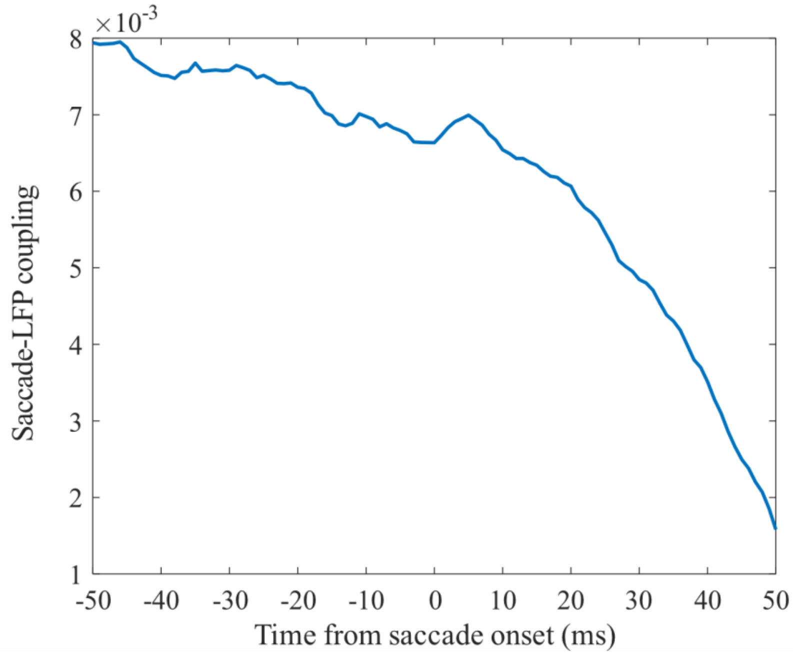

Figure S4. Across-saccade phase similarity for beta LFPs (17-20 Hz) around the saccade onset. Here, for removal of the transient neural response, we subtracted the average saccade-related response (ERP) (computed session-wise) from the neural activity for each saccade. Results indicate that the across-saccade phase similarity is not stronger in the post-saccade period during the very close neighborhood around the saccade onset.

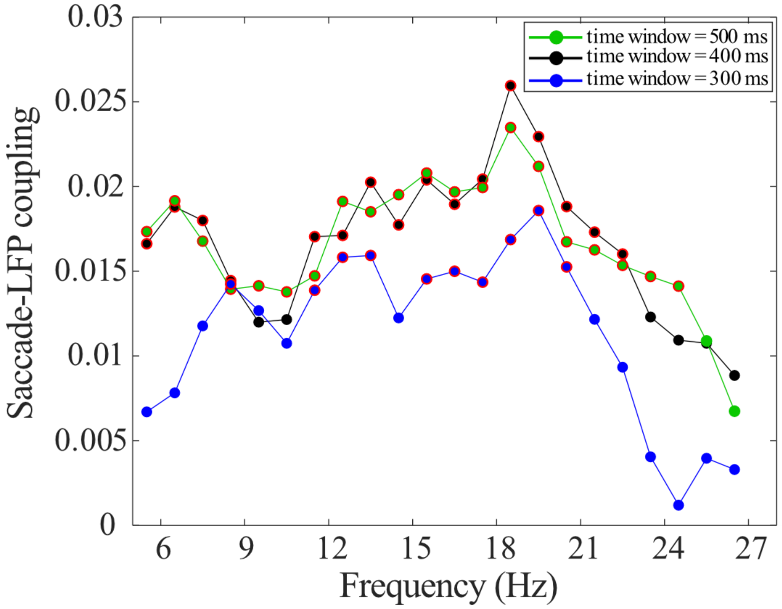

Figure S5. Re-calculation of saccade-phase locking with different temporal smoothing and filter order settings. The across-saccade phase similarity was calculated at saccade onset by considering different analysis windows and filter orders (a 300, 400 and 500 ms interval preceding the saccade onset with filter orders 1301, 1401 and 1501, respectively) of LFPs. Red circles indicate a significant phase similarity.

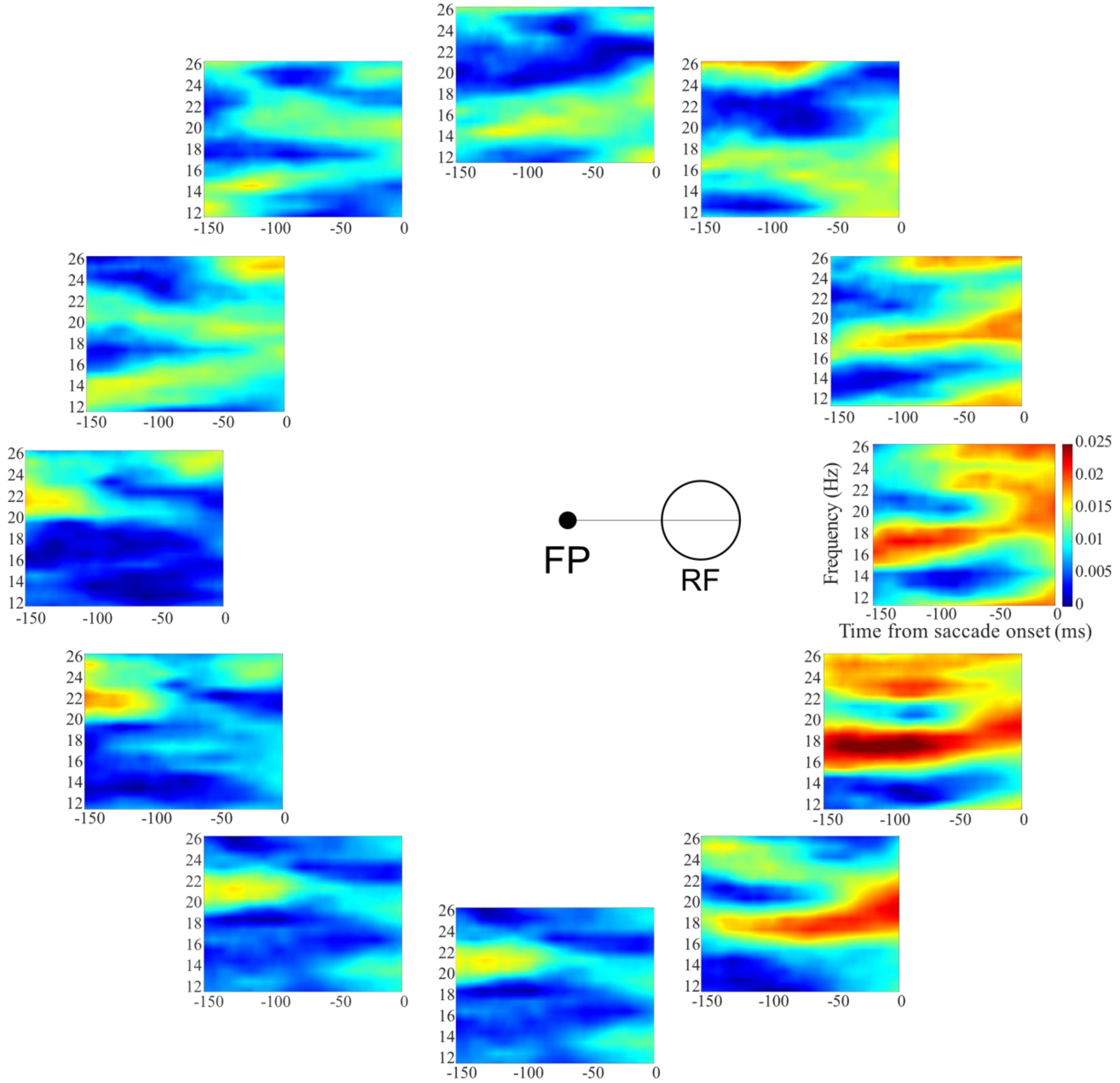

Figure S6. Across-saccade phase similarity for various saccade directions relative to the neuron's RF. Saccades were categorized into 12 circular bins based on their direction relative to the line connecting the FP and the RF (90°-wide bins overlapping by 30°): (-45°, 45°), (-15°, 75°), ..., (-75°, -15°).

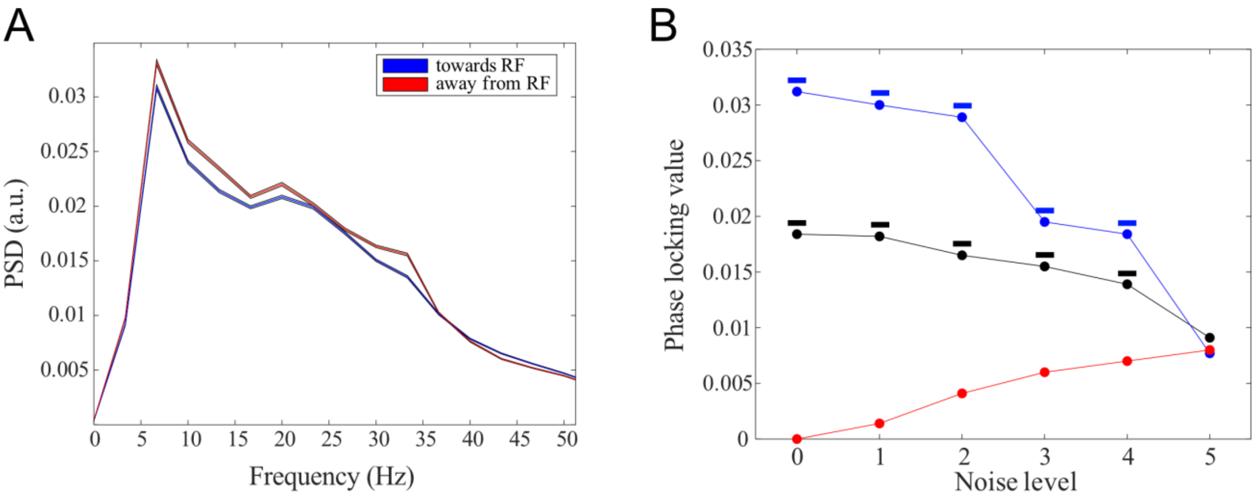

*Figure S7. Direction selectivity of saccade-LFP coupling is not an artefact of the LFP power difference. A) Power spectral density of the pre-saccadic LFP for saccades directed towards the RF (blue) and away from the RF (red). Error bars show the SEM. B) PLV-SNR relationship at different levels of ideal phase-locking. We generated synthetic signals at three different levels of phase-locking (0, 0.018 and 0.031). For each phase-locking level, we generated 16,000 sinusoid signals at 20 Hz with either none of them (for level 0) or a proportional subset of them phase-aligned to each other (for the case of levels 0.018 and 0.031) and afterwards, introduced various levels of noise to the data, thus decreasing the SNR. We then calculated PLV for these simulated data with different noise levels (noise levels 0-5 = original signals + [0, 5, 10, 20, 30, 50] * randn). These results indicate that when phase-locking exists in the original data, SNR and measured PLV are positively correlated.*

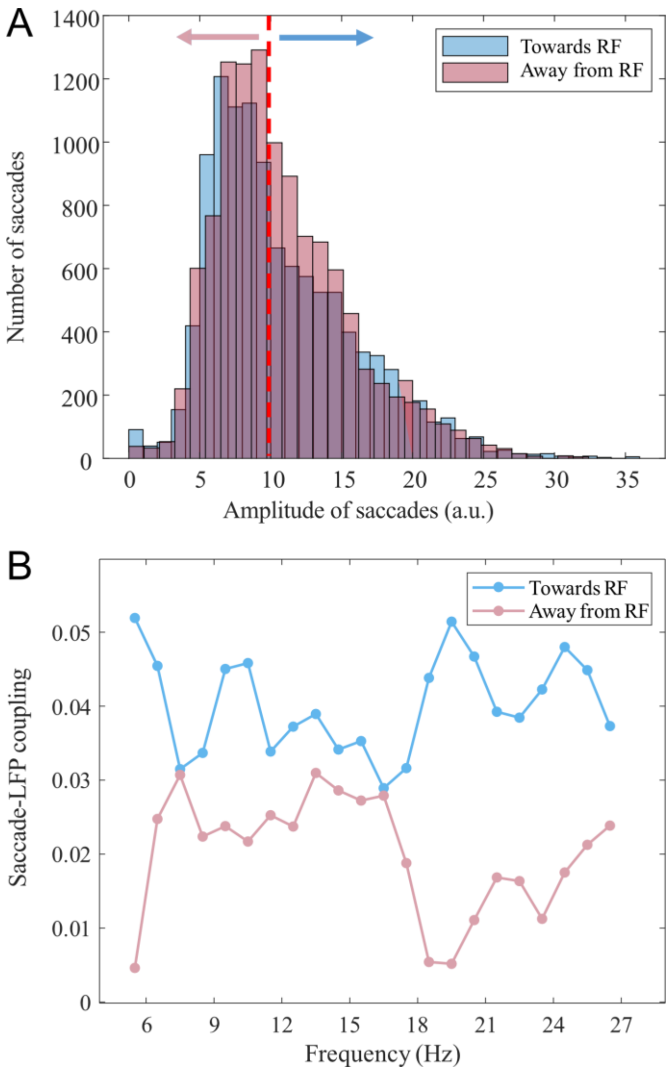

Figure S8. Direction selectivity of saccade-LFP coupling is not due to saccade amplitude differences. A) histogram of the amplitude for saccades towards and away from RF. Saccades towards the RF were significantly shorter than saccades away from the RF (Wilcoxon rank sum test, p<0.05), evoking the question if their difference in saccade LFP coupling may be a side effect of saccade amplitude difference. B) Saccade-LFP coupling measured by PLV for two extreme cases of saccade towards/away with large and small saccade amplitudes (as opposed to the original data). To examine if the direction selectivity of saccade-LFP coupling is due to saccade amplitude differences between saccades towards vs. saccades away from RF, we selected an extreme subset of saccades towards RF with their amplitude larger than 10 and an extreme subset of saccades away from RF with their amplitude smaller than 10. The coupling of saccades to the LFP phase remains large for saccade towards compared to saccade away from RF, suggesting that the direction selectivity of the saccade LFP coupling is not an artefact of the saccade amplitude. Note that all of the frequency points in the saccades towards RF condition have a significant PLV, while for the saccade away condition, this is not the case for any of the frequency points. The threshold 10 was selected to maximize the number of saccades in each subset.

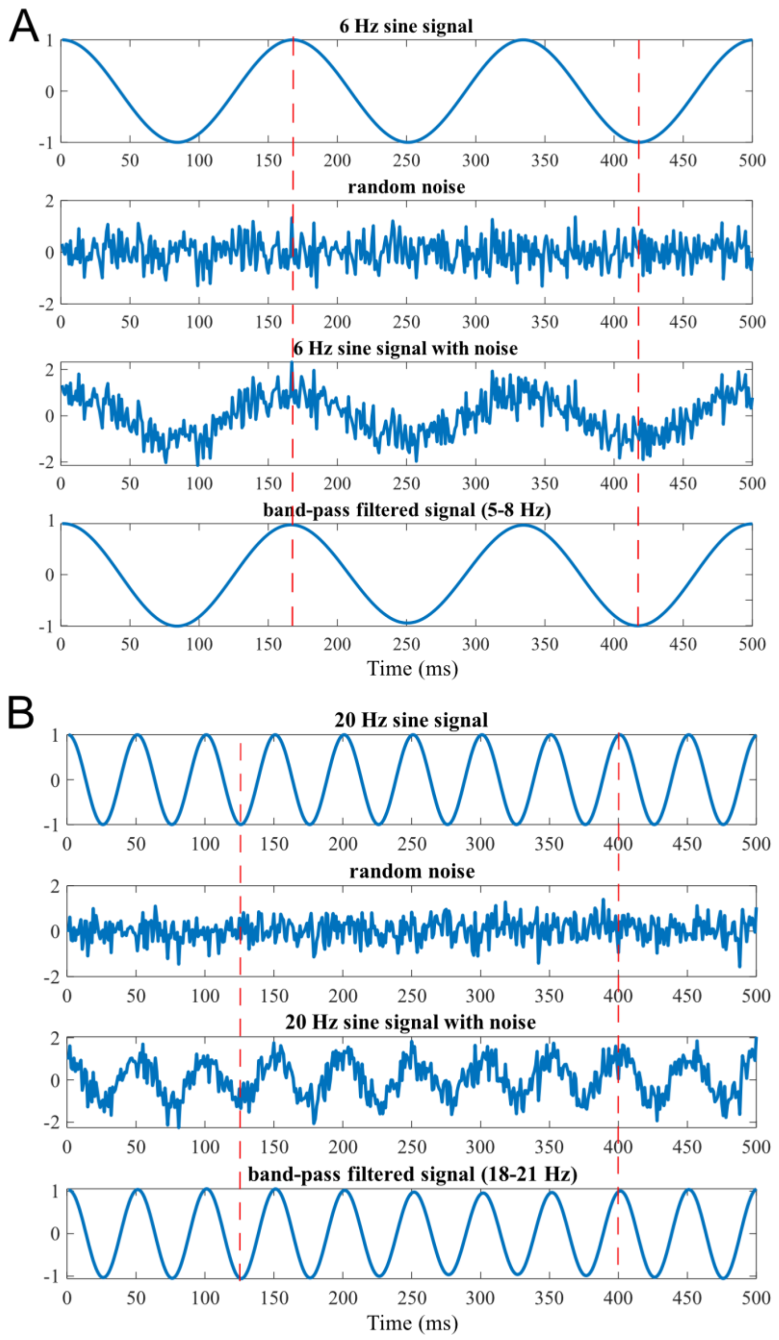

Figure S9. Absence of phase-distortion using our band-pass filtering methods, revealed on simulated data. We generated a sine signal, added normal noise to it, and then band-pass filtered the signal within a sample of target frequency bands (5-8 and 18-21 Hz). Results show that the filtering approach used in this study does not result in phase distortion/delay. A) 6 Hz sine signal (first panel), random normal noise signal (second panel), noise signal added to the signal (third panel), the result of band-pass filtering the synthetic signal within 5-8 Hz (fourth panel), B) 20 Hz sine signal (first panel), random normal noise signal (second panel), noise signal added to the signal (third panel), and the result of band-pass filtering the synthetic signal within 18-21 Hz (fourth panel).

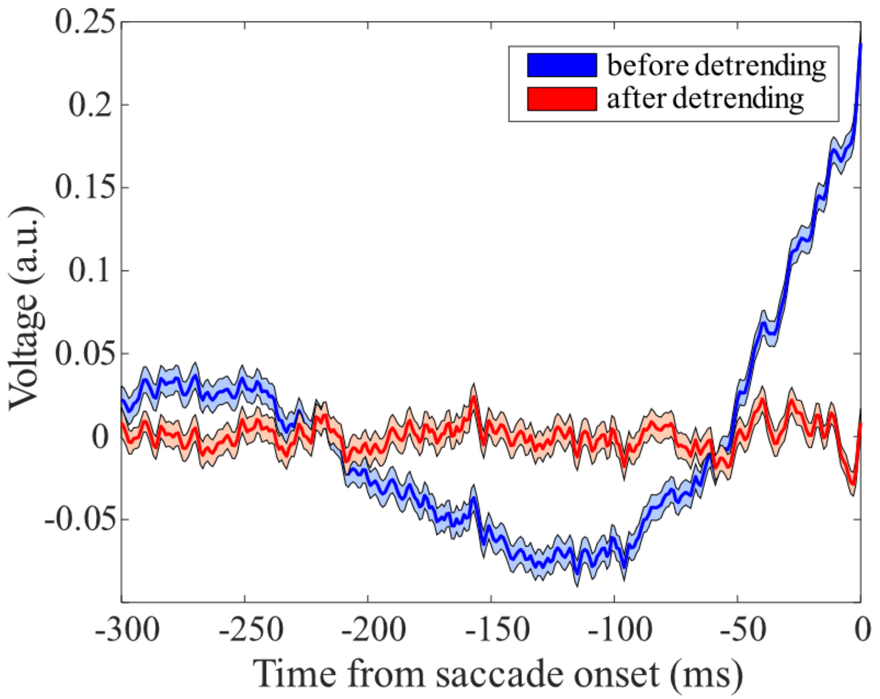

Figure S10. Saccade-triggered LFP across all saccades before (blue) and after (red) removing the transient component before the saccade (by estimating a fourth-degree polynomial trend). Error bars show the SEM.
